# Development of Calcium Carbonate-Based Coatings by the Carbonation of Gamma-C_2_S (γ-C_2_S)

**DOI:** 10.3390/ma15155088

**Published:** 2022-07-22

**Authors:** Ming Lei, Zhichao Liu, Fazhou Wang, Shuguang Hu

**Affiliations:** 1 State Key Laboratory of Silicate Materials for Architectures, Wuhan University of Technology, Wuhan 430070, China; minglei24@whut.edu.cn (M.L.); hsg@whut.edu.cn (S.H.); 2 School of Materials Science and Engineering, Wuhan University of Technology, Wuhan 430070, China

**Keywords:** inorganic coating, γ-C_2_S, carbonation, CO_2_ uptake, porosity, corrosion resistance

## Abstract

A calcium carbonate (CaCO_3_)-based coating with gamma-C_2_S (γ-C_2_S) as the main carbonatable binder is proposed to protect the metal substrate against corrosion in this paper. Here, the morphology and phase assemblage of the coatings are studied by scanning electron microscopy (SEM) and X-ray diffraction (XRD), and the corrosion resistance of the coating is evaluated by electrochemical impedance spectroscopy (EIS) and X-ray photoelectron spectroscopy (XPS). Results show that the carbonated coating has excellent properties in terms of ultraviolet (UV) aging resistance, salt fog resistance, and electrochemical corrosion resistance. The porosity of deposited coating on steel substrates decreases by 47.1% after carbonation due to the compacted calcium carbonate formation, which is recognized as a self-compacting process during carbonation. The coating also exhibits rapid strength development within the first 2 h of carbonation; both the CO_2_ uptake efficiency and degree of carbonation (DOC) can reach more than 95% of the total CO_2_ uptake efficiency and final DOC values. This study provides a novel insight to extend the category of inorganic coating with additional benefits of CO_2_ solidification.

## 1. Introduction

The poor durability of metal-based materials is associated with their inherent defects, as they react easily with moisture, oxygen and other corrosive gases in the atmosphere [1,2,3]. Generally, the metal corrosion is supposed to be mainly divided into electrochemical corrosion [4], chemical corrosion [5] and biological corrosion [6,7], and these corrosions can cause irreversible damage to the metal and significantly reduce its employment durability. An effective remedy is to establish a barrier to block the contact of harmful substances (such as harmful ions, organisms and oxygen) with metal-based materials to achieve the purpose of protection.

Efforts have been taken to alleviate the defects of metal-based materials. Organic/inorganic and hybrid materials have been extensively used to protect metals from corrosion. For instance, organic coatings (e.g., epoxy resin) play an essential role as a physical barrier between the metal surface and the corrosive medium [8,9] due to their high cross-linking density. Silva [10] reported an epoxy resin produced from an agro-industrial byproduct for manufacturing a single component anti-corrosive epoxy coating. Results show that the coating shows high crosslinking and good thermal–mechanical, anti-corrosive, and electrochemical impedance spectroscopy performance with 5 wt% 1-methylimidazole as the accelerator during the exposure period.

However, organic coatings have poor thermal stability and UV resistance under the high energy provided by sunlight, which is greater than the dissociation energy of the organic polymer chain, resulting in molecular chain scission and eventual degradation [11]. In addition, organics can also soften or decompose at a certain temperature (generally ˃100 °C) and are subsequently penetrated by corrosive substances such as oxygen, water and reactive ions [12]. The diffusion of such harmful components into the epoxy coating results in degradation of the organic coating over long exposure times; hydroxyl ions (OH-) generated at the cathode below the coating result in an increase in pH and a decrease in coating adhesion, which accelerates corrosion of the metal below the coating [13]. Those deficiencies severely limit the application range of most organic-containing coatings.

Inorganic or hybrid coatings mitigate the defects of the single organic component coatings. However, the application of an existing inorganic coating such as alkoxysilane, silicon or polysiloxane hybrid coatings is prone to form cracks and microforms during the condensation process [14,15]. These cracks and microforms can cause insufficient mechanical properties and poor protectiveness of the coating. Besides, some carbon-based coatings were also used as a protection barrier in past decades [16,17].

In recent years, attempts have been made to improve the persistence and sustainability of inorganic coatings such as through the utilization of conversion coatings (e.g., phosphate, molybdate, and zirconium conversion coatings) [18,19], which have been employed into the matrix between the organic coating and substrate as a role of an intermediate transition layer. Conversion coatings can improve the epoxy coating’s adhesion to the steel substrate through the surface energy enhancement and changing the surface morphology. Vakili [17] reported an epoxy coating treated by a cerium-based conversion coating to protect the steel substrate. Results show that the epoxy coating’s adhesion to the steel substrate is improved with treatment by Ce and Ce–Zn conversion coatings, and the enhancement in adhesion properties is apparent in the case of Ce–Zn treatment. Bera [20] presented a water-based epoxy–silane hybrid coating to improve corrosion protection and adhesion on galvanized steel. With the grafting of sol-gel to an organic polymer backbone, after adding 1~3 wt% silane into an epoxy polymer backbone, both the anti-corrosive performance and adhesion properties improved significantly when compared to non-grafted epoxy polymer. However, many conversion coatings such as silane or cerium contain toxic and carcinogenic substances, and the use of a conversion coating makes the fabrication process more complicated.

This paper aims to propose a CaCO_3_-based inorganic coating to protect the steel substrate from corrosion, especially for marine environments, which are weakly alkaline (pH 8.0~8.5). Calcium carbonate can exist stably within this range as a protection barrier. γ-dicalcium silicate (γ-C_2_S) is selected as the main carbonatable binder, while the generated calcium carbonate acts as a protective layer. The basic principle is the phase transition in the presence of CO_2_, as shown in Equation (1). The loose γ-C_2_S powders transformed into compact CaCO_3_ including calcite and aragonite with high strength and wear resistance after carbonation, accompanied by the significant decrease of porosity, which imparts the coating with excellent protection for substrates.

(1) $${\mathrm{Ca}}_{2}{\mathrm{SiO}}_{4}+2{\mathrm{CO}}_{2}\overset{H_{2}O}{\Rightarrow }2{\mathrm{CaCO}}_{3}+{\mathrm{SiO}}_{2}$$

This inorganic coating can also be sprayed on the inside of the flue gas (containing CO_2_) pipe to play an additional role in preventing internal corrosion of the pipe. In addition, the composite effect of the inorganic coating with organics (styrene–acrylic emulsion) is also briefly investigated to further extend its application fields. This paper can provide a novel insight into inorganic coatings with the additional benefits of CO_2_ solidification.

## 2. Experimental

### 2.1. Materials and Preparation

γ-C_2_S was prepared by mixing analytical grade Ca(OH)_2_ and SiO_2_ in a stoichiometric ratio of 2:1 with 10% by weight deionized water in an agate pot mill and mixing for 4 h. After drying in an oven at 105 °C for 6 h, the green body was heated at 1400 °C for 3 h at a heating rate of 10 °C min^−1^, and then air-cooled to room temperature in an oven. The density of the pulverized γ-C_2_S powder was 2.92 g cm^−3^ tested by the Le Chatelier Flask method and the median particle size was 14.2 μm measured by laser diffraction particle size analyzer.

The composition of the steel substrate (dimensions of 100 × 100 × 2 mm) is shown in Table 1. The selected steel substrates were abraded from #100, #400, #800 up to #1600 emery paper followed by degreasing using acetone and washed by absolute ethanol. The cleaned steel sheets were dried in an oven at 100 °C, and then cooled at room temperature.

**Figure 1:**
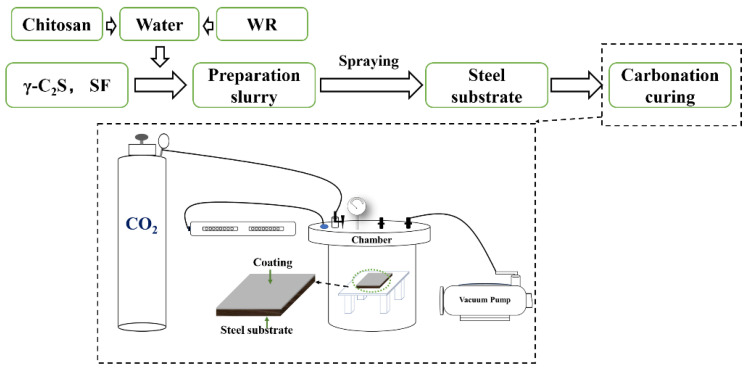
Schematic diagram of preparation process.

The polycarboxylic acid superplasticizer (water reducer, denoted as WR), chitosan, and silica fume (SF) were selected in the mix, whilst the chemical composition of SF is shown in Table 2. The composition of the mix is shown in Table 3. Mixing each component evenly at a stirring speed of 100 r min^−1^, the prepared slurry was then sprayed onto the surface of the steel substrate by a spray gun. The sprayed steel substrate was placed in a stainless-steel chamber. The air in the chamber was exhausted with a vacuum machine, and then CO_2_ with a concentration of 99.99% was introduced into the chamber until the gas pressure was maintained at normal pressure (OP) and 0.1 MPa, and the carbonation time was controlled for 24 h. The specific detail of preparation is shown in Figure 1.

### 2.2. Test Methods

T Haake™ Viscotester™ IQ rheometer (Thermo Scientific™, Dreieich, Germany) was used to measure slurry fluidity at different times after standing, and the rotation speed was controlled from 0 to 100 r min^−1^.

The surface and section morphology of the coating was observed by SEM using an FEI QUANTA FEG 450 ESEM with an accelerating voltage of 5 kV and 10 kV in high vacuum mode.

The porosity of the coating before and after carbonation was characterized by Mercury intrusion porosimetry (AutoPore IV-9500, pore size range: 3 nm–360 mm).

X-ray diffraction (XRD) analysis was measured by using a D8 Advance X-ray diffractometer with the Cu Kα radiation (λ = 1.5405 Å) at 30 kV and 30 mA and the 2-theta value ranging from 10° to 70°. The carbonated coatings were powdered and mixed with 20 wt% corundum powders as an internal standard substance for the quantitative phase analysis. The scanning rate was 2° min^−1^. Rietveld refinement quantitative phase analysis was presented by Jade 6.0 software.

The effect of different carbonation pressures on the corrosion resistance of the coating was characterized by X-ray photoelectron spectroscopy (XPS). All spectra were measured using an ESCALAB 250Xi XPS instrument. A monochromatic A Kα X-ray source was used for all samples, along with pressures in the analysis chamber of 10^−6^~10^−7^ Pa, the sensitivity is about 0.1 at%, the spatial resolution is 100 um, and the analysis depth of X-ray is about 1.5 nm. The source was operated at 15 kV and 20 mA. Curve fitting of the spectra was achieved through a mixture of Gaussian and Lorentzian functions on a Shirley-type background.

The electrochemical impedance spectrum (EIS) was conducted using Auto-lab PGSTAT electrochemical measurement device. Electrochemical tests were performed in a three-electrode corrosion cell. A saturated calomel reference electrode (SCE) and platinum foil were used as reference and counter electrodes, respectively. The steel samples (10 × 10 mm) were fixed at the end of Teflon tube and sealed by epoxy adhesive. The samples were connected with copper wire and adopted as working electrodes for polarization measurements. Electrochemical impedance measurements were performed on the bare steel and coated samples immersed in 300 mL of 3.5 wt% NaCl solution at ambient temperature as a function of immersion time up to 1 h. In addition, the corrosion protection performance of the coated samples was investigated by EIS analysis. The measuring frequency for the EIS analysis ranged from 100 kHz to 10 mHz and the value of peak to zero amplitude was ±10 mV. The electrochemical parameters were obtained using the software package of Autolab workstation. The fitted data of the EIS spectra were obtained from Nova software.

The salt spray test was performed on coated sheets with an artificial configuration of simulated seawater solution at 100% relative humidity at 35 °C according to ASTM B117. The samples were checked every 24 h and images were recorded to validate the EIS results.

## 3. Results

### 3.1. Characterization of Slurry

**Figure 2:**
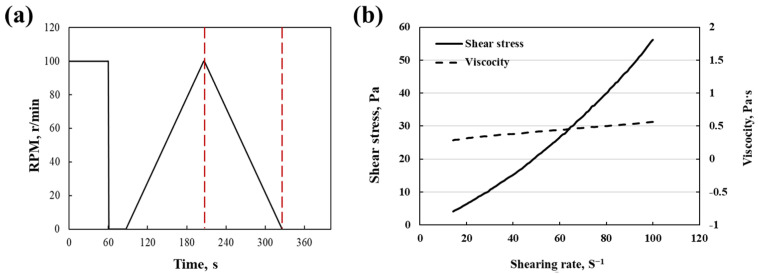
(**a**) Shearing velocity at different times and (**b**) slurry viscosity at different shearing rates.

The viscosity and shear stress of the mix are shown in Figure 2. The rotational speed of the stirrer is from 0 to 100 r min^−1^ (between the red dotted areas in Figure 2a). As can be seen, the shear stress gradually increases with the increase of shear rate, and the viscosity of the slurry remains at a stable value (about 0.45 Pa·s), which indicates the fine stability and uniformity of the slurry (Figure 2b). Higher fluidity and lower viscosity of slurry are essential for slurries that need to be sprayed through a spray gun, higher viscosity can result in the slurry blocking the nozzle of the spray gun during spraying. On the contrary, too low viscosity is not conducive to the uniform dispersion of the slurry during spraying, and a higher water-to-binder ratio can lead to the existence of more interfacial transition zones and reduce the strength of the carbonated coating [21,22].

### 3.2. Phase Assemblage, Morphology, and Porosity

**Figure 3:**
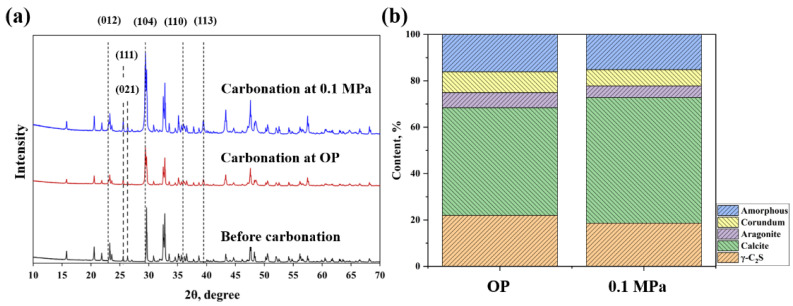
(**a**) XRD pattern and (**b**) the phase assemblage of carbonated coating.

The phase assemblage of coating is characterized by XRD. The carbonation products of coatings with different carbonation pressures are shown in Figure 3. It can be seen that the carbonation products of the coating are mainly composed of calcite and a trace amount of aragonite. The silica fume could not be detected by XRD due to its amorphous characteristic. Quantitative analysis of carbonated components is measured by the Rietveld method [23]. With the increase of carbonation pressure, the production of calcite is further promoted, while the increase of the amount of aragonite is not obvious (Figure 3b). As can be seen, (012), (104), (110), (113) are typical characteristic peaks of calcite, and (111), (021) are typical characteristic peaks of aragonite. The intensities of these characteristic peaks are increased and accompanied by a decrease in the intensity of γ-C_2_S. This is mainly due to the fact that the enhancement of CO_2_ pressure can promote the dissolution of calcium ions (Ca^2+^), which facilitates calcium carbonate formation and the reaction promotion [24,25].

In addition, it is worth noting that whether the carbonation pressure is ordinary pressure (OP) or 0.1 MPa, there is still about 20% of uncarbonated γ-C_2_S existing in the sample. This is related to the fact [24] that the carbonation reaction is a process from the outside to the inside; in the initial carbonation stage, the generated calcium carbonate and amorphous silica gel can gradually cover the outer layer of the particles, while the uncarbonated γ-C_2_S in the inner layer finds it difficult to participate in the following carbonation reaction. Furthermore, due to the low thickness of the coating, it generates a greater amount of carbonation products such as calcites (near 42%) under the same carbonation conditions when compared with the γ-C_2_S compacts prepared by press forming (near 30%) [25]. Besides, comparing the products under different carbonation pressures, with the increase of carbonation pressure (from OP to 0.1 MPa), the amount of aragonite decreases slightly. This is related to the fact that in the early stage of carbonation, higher carbonation pressure is more conducive to the formation of calcite compared to aragonite [26].

**Figure 4:**
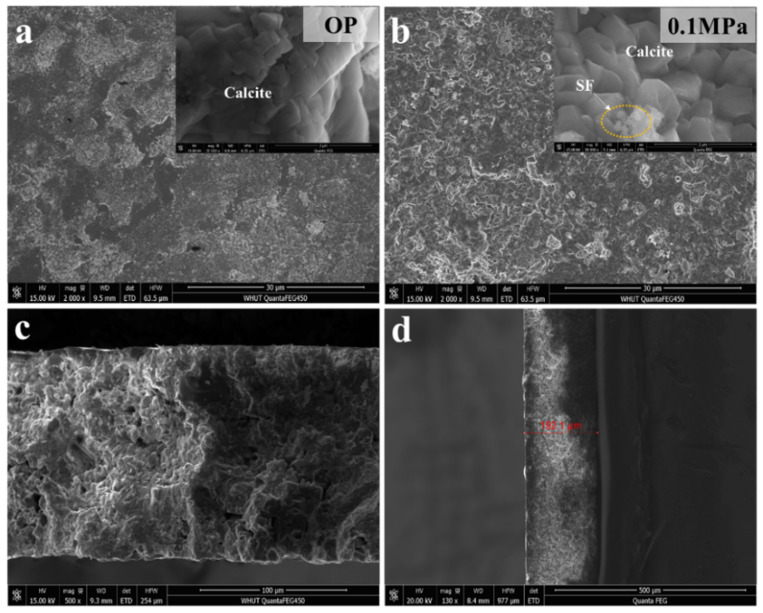
SEM images of the carbonated coatings. (**a**,**b**) the coatings after carbonation with different CO_2_ pressures. (**c**,**d**) the cross section of the coatings (0.1 MPa) under different multiples.

Figure 4 shows the SEM image of the coatings after carbonation with different CO_2_ pressures. The main product of carbonated coating is cube-like calcite (Figure 4a,b), which is consistent with the results of XRD. The sporadic silica fume can also be observed in Figure 4b, which further fills the pores that exist in the interzone between crystal particles. Regardless of carbonation pressure, the generated CaCO_3_ crystals are very dense with a large amount of calcium aggregated together to form a compact protection layer under the microscopic morphology. In addition, the porosity of the coating is very small which, along with the mutual independence of the pores (Figure 4c,d), makes it difficult for the corrosion solution to reach the bottom of the coating and contact the covered steel substrate.

**Figure 5:**
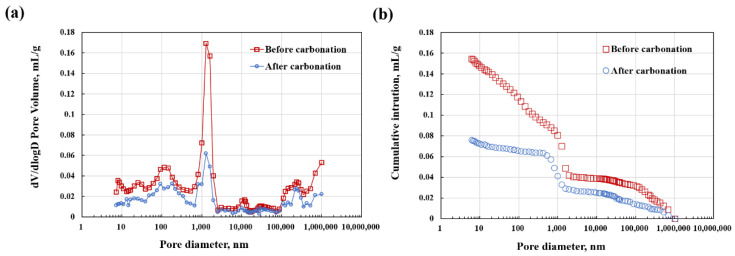
(**a**) Pore size distribution and (**b**) cumulative pore volume of the coating before and after carbonation.

The pore size distribution and cumulative pore volume of the coating before and after carbonation measured by Mercury intrusion porosimetry (MIP) are presented in Figure 5. As can be seen, the distribution in each pore size interval and total cumulative pore volume of the coating is significantly reduced after carbonation. The most probable pore size decreases from 1260 nm to 1080 nm while the cumulative pore volume of the coating decreases from 0.17 mL/g to 0.09 mL/g, an almost 47.1% reduction of porosity. Previous studies show that the carbonation is recognized as a voluminous reaction [24,25]. The generated calcium carbonates can fill the present micropores with the progress of the reaction, which can also be demonstrated by a pore size change of less than 1 μm, resulting in the carbonated coating with a dense structure that is capable of being employed as an anti-corrosion production.

### 3.3. Corrosion Resistance of Coating

**Figure 6:**
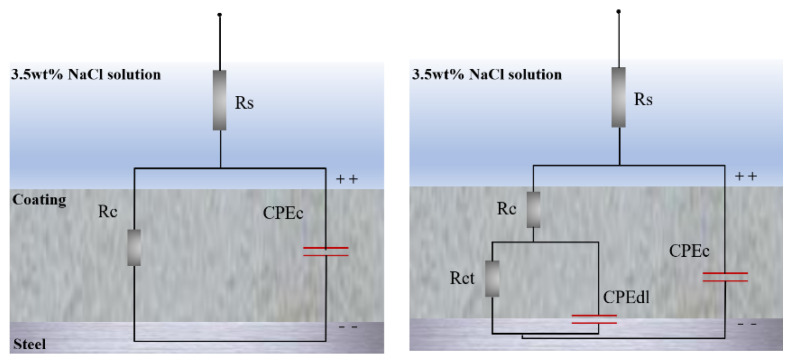
Equivalent circuits for modeling experimental impedance data.

EIS is used to evaluate the corrosion-resistant performance of the coating. The bare steel substrate and coated steel substrate with calcium carbonate are selected to compare at room temperature after the open circuit potential (OCP) is stable. The corresponding impedance spectrum (CIS) is shown in Figure 6. To simulate EIS data, two different electronic equivalent circuits (EECs) are considered [27,28]. In these two models, a constant phase element (CPE) is used instead of the ideal capacitor on the basis of the porosity and unevenness of the formed layer. In the proposed EEC, Rs is the solution resistance, Rct is the charge transfer resistance, and CPEc is the double-layer non-ideal capacitor.

**Figure 7:**
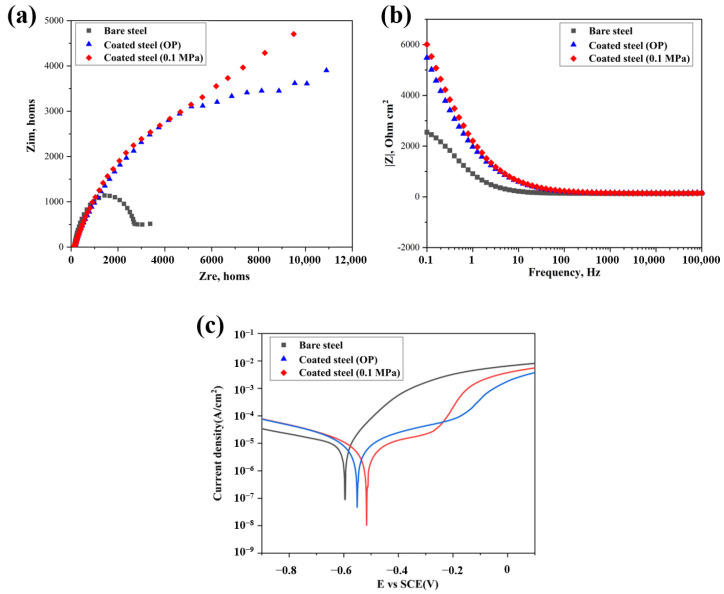
(**a**) Nyquist plots, (**b**) Bode plots and (**c**) Potentiodynamic polarization curves of bare steel substrate and coated steel substrate after carbonation.

Figure 7a shows the Nyquist plots of the bare steel and the coated steel immersed in 3.5 wt% NaCl solution, respectively. The electrochemical impedance of the coated substrate is significantly higher than that of the untreated bare steel after fitting, irrespective of the carbonation pressure (OP or 0.1 MPa), and it increases with the increase of carbonation pressure. This can also be observed from Bode plots (Figure 7b), due to the existence of the passivation film on the steel surface. The corrosion resistance of the steel base surface is about 2500 in the initial stage, almost 2.4 times lower than that of the coated steel. The compactness of the coating and the disconnection of the micropores greatly improves the impedance. Even if there are some micropores, their non-connectivity makes it difficult to provide a path for the diffusion of the corrosive electrolyte to make contact with the steel substrate; the protected steel substrate with calcium carbonate coating is difficult to delaminate, and generate corrosion products. As the carbonation pressure increases, the resistance of the coating also increases, which shows a better anti-corrosion trend. In the electrochemical workstation, the potentiodynamic polarization provides strong support for the corrosion resistance of the coating. The Tafel curve obtained under the corrosive electrolyte is shown in Figure 7c. Compared with a natural passivation film on the surface of the substrate, the corrosion current density of the calcium carbonate coating is reduced by an order of magnitude. This is due to the fact that the coating prevents the NaCl solution from reaching the interface and minimizes the creation of surface corrosion sites.

**Figure 8:**
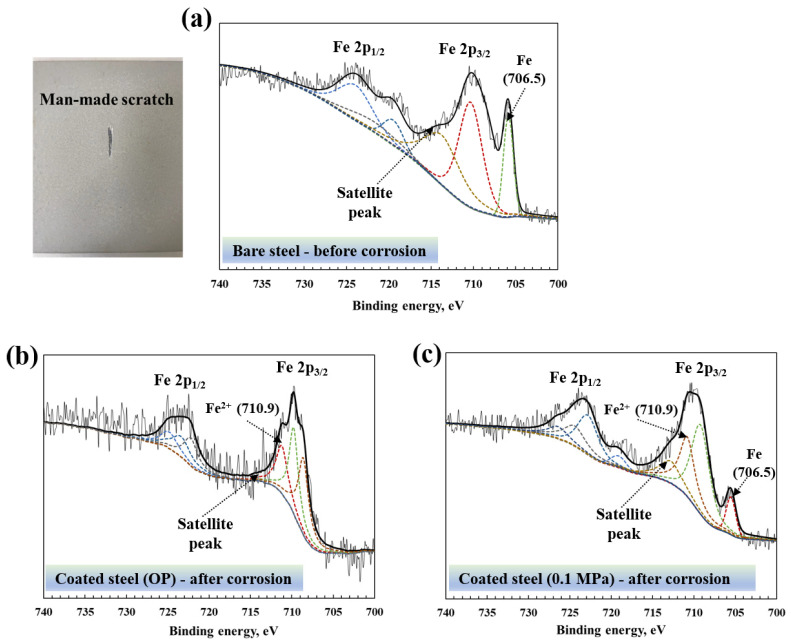
XPS spectrum of the (**a**) bare steel before accelerated corrosion and (**b**,**c**) the coated steel cured with different carbonation pressure after corrosion.

XPS is also used to compare the discrepancy in corrosion resistance of coatings cured under different CO_2_ pressures. An accelerated corrosion experiment (man-made scratch) is performed to detect the corrosion degree of the steel substrate, which experienced the same corrosion duration. The deconvolution of multiple peaks of Fe at the interface between the coating and the steel substrate to obtain a high-resolution XPS spectrum. The diagram of bare steel before accelerated corrosion is shown in Figure 8a, the obvious Fe peak with 706.5 eV binding energy can be observed, and the Fe 2p_1/2_ and Fe 2p_3/2_ with its satellite peak can also be detected at the typical Fe binding energy at 700–740 eV [29,30]. After accelerated corrosion, the coated steel substrate with OP shows that the binding energy of Fe at 706.5 eV (Fe 2p_3/2_) disappears during the corrosion process, accompanied by the gradual generation of the Fe^2+^ peak at 710.9 eV (Figure 8b). In the meantime, the coated steel substrate with 0.1 MPa still exists the peak of Fe at 706.5 eV although the signal intensity is weakened and the peak intensity is reduced, and the Fe^2+^ peak with binding energy at 710.9 eV has just begun to appear (Figure 8c). The results of XPS demonstrate that the coating cured at higher carbonation pressure has higher corrosion resistance.

**Figure 9:**
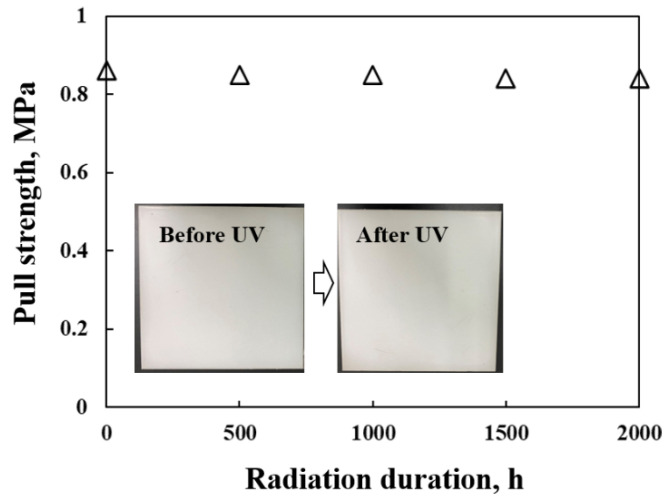
The pull strength of the coating after UV radiation with different duration.

The organic or hybrid coatings have poor performance in anti-UV aging degradation, owing to the fact that the molecular chains of organic molecules are easy to break into short chains or even decompose under a UV-accelerated aging environment, which results in structural changes and ultimately affects the employment performance [31,32]. Here, the anti-UV aging degradation of the coating is explored, the coated steel is tested in an artificially accelerated UV aging box, the light source is a quartz lamp, the wavelength is 253 nm, the temperature is 60 °C, and the relative humidity is 50% ± 5%. After irradiation for 30 days, there is no obvious change in the coating surface, and the pull strength of the coating can still remain above 95% of the total strength (Figure 9). The previous study reveals that the calcium carbonate forms such as calcite can increase the mechanical strength even when the temperature is enhanced to 500 °C [33]. This may be attributed to the effect of crystal transformation at this temperature, which reduces the porosity of the matrix and increases the density simultaneously.

**Figure 10:**
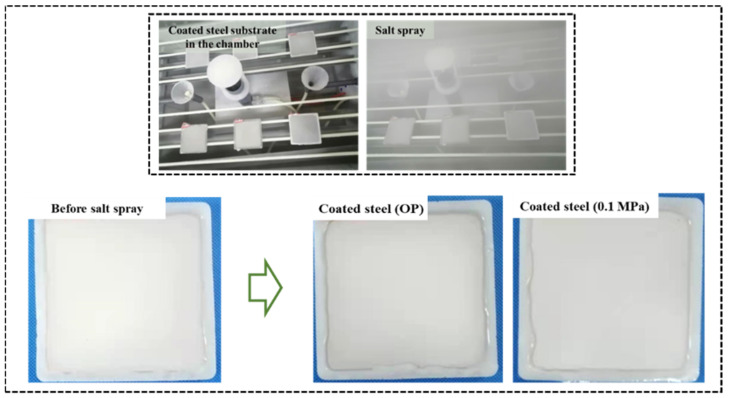
Salt spray test of the coated steel substrate.

The salt spray test as a conventional index of coating is carried out to evaluate the durability of the calcium carbonate coating. The artificial seawater (Table 4) is prepared to be continuously sprayed in an ASTM B117 environmental chamber. The perimeter and bottom of the specimens are coated with pure epoxy resin to achieve the purpose of one-sided erosion. The image of the coated steel after salt spray for 1200 h is shown in Figure 10. The results show that there is no rust on the surface of the coating under different carbonation pressures even if the color of the coating is deepened, which is associated with prolonged salt deposition.

### 3.4. Application Prospect

The CO_2_ absorption capacity of the carbonated coating is very important for its sustainable application. The mass change of the coating after carbonation is a way to characterize the amount of CO_2_ uptake. The CO_2_ absorption here is obtained by the mass change of the matrix before and after carbonation under the premise of complete drying, so as to eliminate the water loss interference in the calculation. The amount of CO_2_ absorbed by the coating is estimated by the following Equation (2).

(2) $${\mathrm{CO}}_{2}\;\mathrm{uptake}\;=\frac{M_{B}-M_{A}}{M_{A}}$$

where the M_A_ and M_B_ are the absolute dry mass of carbonated coating before and after carbonation.

**Figure 11:**
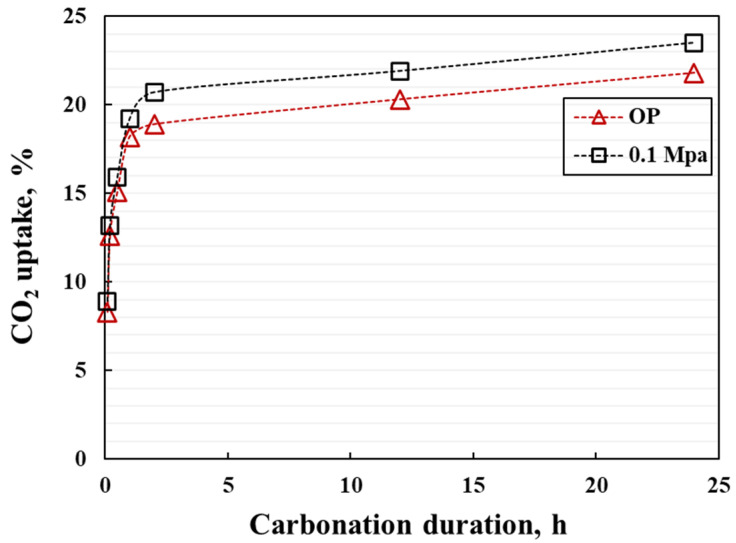
CO_2_ uptake rate of the coating during the carbonation.

The CO_2_ uptake efficiency of the coating during carbonation is shown in Figure 11. It can be seen that the coatings under different carbonation pressures have the same trend of CO_2_ absorption efficiency, and within the first 2 h, the CO_2_ uptake efficiency increases rapidly, reaching more than 95% of the final CO_2_ uptake efficiency. After 2 h of carbonation duration, the CO_2_ absorption efficiency tends to be stable, and there is no significant increase even if it is extended to 24 h. This demonstrates that the thin coatings exhibit rapid strength development, which greatly improves the manufactural efficiency.

Due to that fact that H_2_O and CO_2_ are the only species released by the carbonation product during heating, the different release temperatures are usually utilized to determine the degree of carbonation (DOC) of γ-C_2_S. The decomposition of CaCO_3_ from 400 °C to 800 °C, and the mass loss of the carbonated coating in this temperature interval are only due to the release of CO_2_. According to the Equations (3) and (4) for calculating the DOC of carbonated costing.

(3) $$2{\mathrm{CaCO}}_{3}+{\mathrm{SiO}}_{2}\overset{\Delta }{\Rightarrow }2\mathrm{CaO}+{\mathrm{SiO}}_{2}+2{\mathrm{CO}}_{2}↑$$

The conversion between DOC and mass loss can be calculated as follows:

(4) $$L=m\;\times \;D\;\times \;\frac{{2\;\times \;M}_{{\mathrm{CO}}_{2}}}{M_{\gamma -C2S}}/\;(m+m\;\times \;D\;\times \;\frac{{2\;\times \;M}_{{\mathrm{CO}}_{2}}}{M_{\gamma -C_{2}S}})$$

where L is mass loss of the sample heated from 400 °C to 800 °C, m is the initial mass of the carbonated coating before carbonation, D represents the DOC, ${\;M}_{{\mathrm{CO}}_{2}}$ and $M_{\gamma -C_{2}S}$ are the molar mass of CO_2_ and γ-C_2_S, respectively.

**Figure 12:**
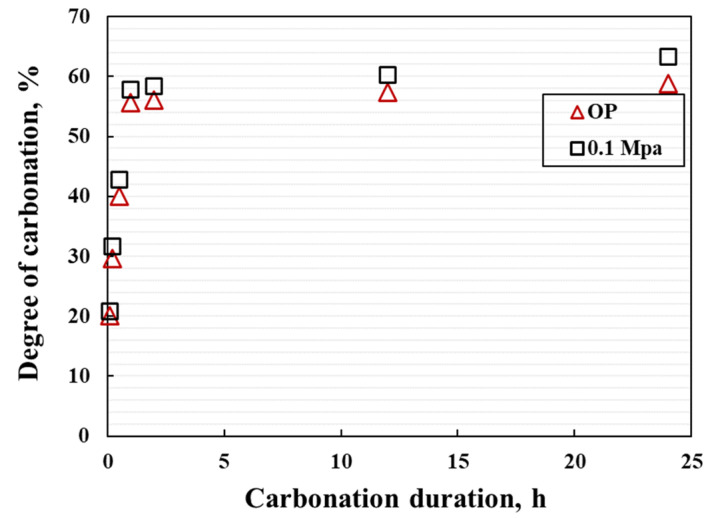
DOC variation of the coating during the carbonation.

It can be seen that DOC gradually increases with the prolongation of carbonation duration, which shows a similar trend to CO_2_ absorption (Figure 12). In 2 h, DOC has reached more than 55% regardless of the CO_2_ pressure, and more than 95% of the total DOC occurs within 2 h.

**Figure 13:**
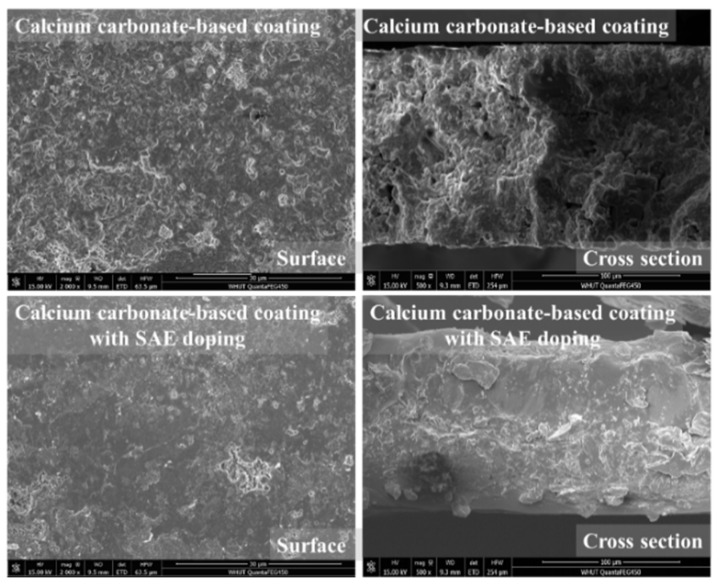
SEM images of surface and cross section of pure inorganic coating and 10 wt% SAE doped coating.

Besides, this calcium carbonate-based coating can be compounded with organic matter to form a composite coating. Here, the styrene–acrylic emulsion (SAE) is selected as an organic additive to dope into the mix. As shown in Figure 13, when the doping amount of SAE is 10 wt%, it can be observed that the surface and cross-section of the composite coating are significantly refined. The organic additive can nearly cover the surface of the inorganic coating after the organic film formation, which implies the composite coating can also be applied in the organic coating layer application field.

## 4. Conclusions

The high carbonation reactivity of γ-C_2_S makes it a promising CO_2_-activated binders. This study aims to propose a calcium carbonate-based coating to protect steel substrates from corrosion by accelerated carbonation of γ-C_2_S-based mix. The main conclusions are as follows.

(1) Calcite and aragonite are the main products of γ-C_2_S-based coatings after carbonation.
(2) The disconnection of pores is accompanied by a significant decrease in porosity after carbonation, making it difficult for corrosive substances to reach the interface between the coating and the steel substrate for erosion.
(3) The corrosion resistance of the coating is significantly improved after carbonation. With the increase of carbonation pressure, the corrosion resistance of the coating is also gradually enhanced, which is mainly due to the fact that the higher CO_2_ pressure increases the production of calcite and aragonite to form a denser matrix, which can also be observed by XPS results.
(4) The carbonated calcium carbonate-based coating has superior resistance of anti-UV aging degradation, and salt spray erosion due to the high cross linked calcium carbonate crystal and the filling effect of added silica fume.
(5) The coatings exhibit rapid strength development, within the first 2 h of carbonation. Both the CO_2_ uptake efficiency and degree of carbonation (DOC) can reach more than 95% of the total CO_2_ uptake efficiency and final DOC values.
(6) The calcium carbonate-based coating can also be compounded with organics (such as styrene–acrylic emulsion) to form a composite coating. The refined hybrid coating significantly improves its densification to further extend the application field of this inorganic coating.

## Figures and Tables

**Table 1:** Chemical composition of steel substrate.

Elements	Fe	C	Si	Mn	P	S	Al
wt%	99.27	0.08	0.26	0.31	0.03	0.07	0.01

**Table 2:** Chemical composition of silica fume.

Elements	SiO_2_	CaO	Al_2_O_3_	K_2_O	MgO	MnO	LOI
wt%	94.58	0.40	0.52	1.06	0.65	0.65	1.70

**Table 3:** Mixture proportion.

Composition	γ-C_2_S	SF	H_2_O	Chitosan	WR
Mass, g	80	10	30	1.2	2

**Table 4:** Concentrations of inorganic salts in artificial seawater.

Composition	NaCl	MgCl_2_·6H_2_O	CaCl_2_·2H_2_O	Na_2_SO4	KCl	NaHCO_3_
Concentration (g L^−1^)	25.1	11.2	1.5	4.1	0.65	0.2

## Data Availability

Not applicable.
